# Intermittent hypoxic conditioning restores neurological dysfunction of mice induced by long‐term hypoxia

**DOI:** 10.1111/cns.13996

**Published:** 2022-11-19

**Authors:** Gaifen Li, Yuying Guan, Yakun Gu, Mengyuan Guo, Wei Ma, Qianqian Shao, Jia Liu, Xunming Ji

**Affiliations:** ^1^ Beijing Institute of Brain Disorders, Laboratory of Brain Disorders, Ministry of Science and Technology, Collaborative Innovation Center for Brain Disorders, Beijing Advanced Innovation Center for Big Data‐based Precision Medicine Capital Medical University Beijing China; ^2^ Department of Neurosurgery, Xuanwu Hospital Capital Medical University Beijing China

**Keywords:** conditioning, hypoxia, inflammation, neurogenesis, neurological function

## Abstract

**Background:**

Central nervous system diseases are associated with hypoxia, which usually cause irreversible nerve damage, but the underlying mechanism is unclear and effective intervention strategies are lacking. This study was designed to explore the mechanism and treatment strategy of hypoxia‐induced nerve injury.

**Methods:**

In this study, 13% O_2_ was used to treat mice for 0, 1, 3 7, and 14 days, Morris water maze and other animal behavior experiments were used to evaluate the neurological function of mice. TUNEL, BrdU, PCNA, DCX, and SOX2 staining were used to observe the apoptosis and proliferation of mouse neurons. RT‐PCR and Iba1 staining were used to evaluate the release of inflammatory factors IL‐1β, IL‐6, and TNF‐α and the activation of microglia.

**Results:**

Short‐term hypoxia promotes neurogenesis, while long‐term hypoxia inhibits neurogenesis. The changes in hypoxia‐induced neurogenesis were positively correlated with neurological functions, but negatively correlated with apoptosis. Moreover, intermittent hypoxic conditioning restored long‐term hypoxia‐induced neurological dysfunction by promoting neural stem cell generation and inhibiting the release of inflammatory factors IL‐1β, IL‐6, and TNF‐α and the activation of microglia.

**Conclusion:**

Hypoxia promoted neurogenesis in a time‐dependent manner, and intermittent hypoxic conditioning exerted a neuroprotective effect through promoting neural stem cell generation and suppressing inflammation induced by long‐term hypoxia stress, which provided a novel concept to develop a treatment for hypoxia‐related brain injury.

## Graphical abstract

Intermittent hypoxia adaptation restores behavioral deficits induced by long‐term hypoxia. (A) Pattern of intermittent hypoxia, 5 min 13% O_2_ and 5 min 21% O_2_ in total 10 cycles; (B) Intermittent hypoxia promoted increased flux in mice; (C) Intermittent hypoxia promoted the increase of SO_2_% in mice; (D) Schematic representation of the experimental design. The four intermittent hypoxic mode phase adaptations include Mode①: 5 min 7% O_2_ and 5 min 13% O_2_, a total of 10 cycles. Mode②: 5 min 10% O_2_ and 5 min 13% O_2_, a total of 10 cycles. Mode③: 5 min 21% O_2_ and 5 min 13% O_2_, a total of 10 cycles. Mode④: 5 min 30% O_2_ and 5 min 13% O_2_, a total of 10 cycles. Behavioral tests were performed at 0, 7, and 14 d of hypoxia treatment. (E) The recordings of mice duration on the rotarod test. (F) The discrimination index of mice in novel object recognition. (G, H) The latency and the crossing numbers of mice were recorded in the Morris water maze test. (I, J): Comparison of the effects of four modes of intermittent hypoxia adaptation to alleviate cognitive impairment induced by long‐term hypoxia. Data are expressed as the mean ± SEM, *n* = 10; *p < 0.05; **p < 0.01 versus the Con group. ^#^ *p* < 0.05 versus the H14d group.

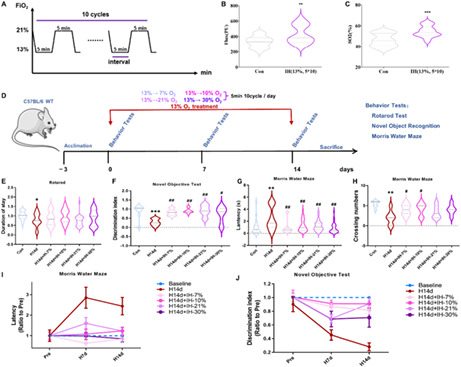

## 1 INTRODUCTION

Oxygen (O_2_) is essential for bodily tissues, and hypoxia usually causes transient or irreversible tissue damage and dysfunction.^1^ Environmental hypoxia is common in aerospace activities, deep sea diving, mountain climbing, and plateau travel, and it usually increases the probability of body or organ damage.^2^ As the most sensitive organ to hypoxia, the brain undergoes a series of response processes when exposed to hypoxia.^3^ Short‐term or mild hypoxia triggers hypoxic responses or conditioning mechanisms in the brain, while long‐term or severe hypoxia often causes irreversible brain damage and neurological dysfunction.^4^, ^5^ However, the differences in hypoxic responses in early hypoxia and the hypoxic damage mechanism in late hypoxia of the brain remain unclear. In addition, the altitude of Lhasa, Tibet, is about 3700 meters (the oxygen concentration is about 13%), which is a common altitude in China and even the whole world.^6^, ^7^ Due to work or other reasons, many people need to go to Lhasa, Tibet, and other places for short‐term or long‐term stays. Hence, understanding the regularity and mechanism about hypoxia and hypoxic responses is important for developing effective intervention strategies for hypoxia‐related brain injury.

Neurons are usually long‐lived cells, although neurogenesis is especially important for neurological function in adulthood.^8^, ^9^ Adult neurogenesis mainly occurs in the dentate gyrus (DG) in hippocampus.^10^ Neurogenesis plays an important role in the maintenance and repair of neurological function, and abnormal changes in neurogenesis are involved in the development of various neurological diseases.^11^ Hypoxia has multiple regulatory effects on neurogenesis, and previous studies reported that hypoxia promoted hippocampal neurogenesis and improved the cognitive function of depressed rats, while other studies reported that hypoxia led to cognitive and behavioral deficits in sleep apnea by inhibiting neurogenesis.^12^, ^13^ Stem cells have the characteristics of self‐renewal and pluripotency, and their main role is to maintain the stability of the tissue environment. They can be activated to proliferate and differentiate into the required type of cells such as neuron, upon the loss of cells or injury to the tissue.^14^ Therefore, it is important to characterize the regulation of hypoxia on neurogenesis and stem cells.

A number of studies have found that intermittent hypoxia had a protective effect on the central nervous system.^15^ Intermittent hypoxia includes preconditioning, phase conditioning, and postconditioning. Intermittent hypoxic preconditioning is the practice of providing hypoxic training in advance, usually for prevention.^16^ Intermittent hypoxic conditioning refers to the administration of lower oxygen concentrations during periods of hypoxia, which can be used for prevention and treatment.^17^ Intermittent hypoxic postconditioning refers to treatment with lower oxygen concentrations after injury, which is usually used for treatment and has less significance than the first two methods.^18^ Due to work requirements and time constraints, people do not have enough time for intermittent hypoxic preconditioning, so in this study we observed the neuroprotective effects of intermittent hypoxic conditioning, to provide a new theoretical basis for the treatment of long‐term hypoxia‐induced brain injury. Hypoxia triggers nerve damage through inflammatory stimulation, eventually leading to neurological dysfunctions.^19^, ^20^ During neuroinflammation, the production of pro‐inflammatory factors including interleukin‐1 (IL‐1), interleukin‐6 (IL‐6), and tumor necrosis factor (TNF) are increased.^21^, ^22^ Microglia are a type of glial cell, the brain equivalent of macrophages, that can clear the brain of damaged nerves, plaques, and infectious substances.^23^, ^24^ However, overactivation of microglia can cause neurotoxicity, leading to production of pro‐inflammatory factors.^25^, ^26^

In the study, we found that there was a bidirectional regulation of hypoxia on neurogenesis, accompanied by changes in neurological functions. Long‐term hypoxia induced nerve damage in mice by inducing neural stem cell depletion leading to neurogenesis disorders. We also found that intermittent hypoxic conditioning restored long‐term hypoxia‐induced neurological dysfunctions by inhibiting inflammation. Our study results, therefore, provided a potential theoretical basis for hypoxia‐induced nerve damage.

## 2 MATERIALS AND METHODS

### 2.1 Animals

Two‐month‐old C57BL/6 mice (weighing 20–25 g) were purchased from Beijing Vital River Laboratory (Beijing, China). All animals were placed in groups of four or five, and were maintained under standard laboratory conditions (12/12‐h light/dark cycle, 23 ± 2°C, with ad libitum access to food and water). All animal experiments were approved by the Animal Care and Use Committee of the Institute of Animal Management, Capital Medical University (permit no. AEEI‐2021‐058), and conducted in accordance with ethical requirements.

### 2.2 Hypoxia treatment

#### 2.2.1 Long‐term hypoxia treatment

**FIGURE 1:**
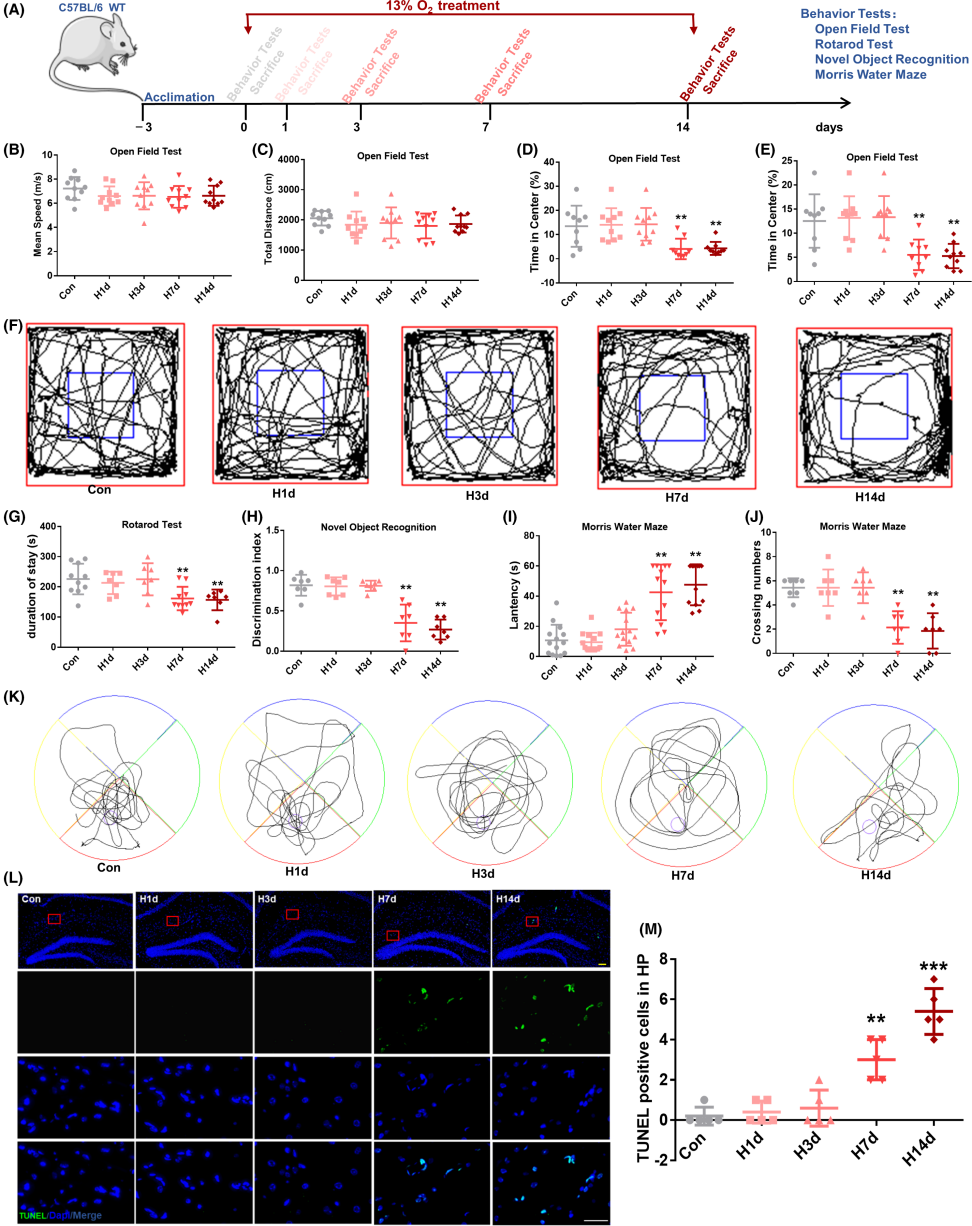
Experimental design and the effects of hypoxia on mice behavior and hippocampus. (A) Schematic representation of the experimental design. Behavioral tests were performed at 0, 1, 3, 7, and 14 d of hypoxia treatment. (B, C) Mean speed and distance in the total zone were recorded in the OFT. (D, E) Time and distance in the center (%) were recorded in the OFT. (F) Tracks of mice in the OFT. (G) The recording of mice duration in the rotarod test. (H) The discrimination index of mice in novel object recognition. (I, J) The latency of mice and the number of times they crossed the platform were recorded using the Morris water maze test. (K) Tracks of mice in the MWM. (L, M) TUNEL staining and the count of TUNEL‐positive cells in hippocampus, Bar = 50 μm. All data are expressed as the mean ± SD; *p < 0.05; **p < 0.01 versus the Control (Con) group.

Research has reported that long‐term exposure to high altitude impaired spatial working memory.^27^ To determine if living at high altitudes resulted in memory impairment after entering Lhasa, the low oxygen concentration in our experiment was 13% O_2_ (simulating the oxygen concentration at the Lhasa, Tibet altitude). We treated mice with 13% O_2_ for 1 d, 3 d, 7 d, and 14 d (Figure 1A) to identify correlations between hypoxia and neurological function. Control animals (treated with 21% O_2_) were left alone, except for regular cleaning of their cages.

#### 2.2.2 Intermittent hypoxia treatment

Intermittent hypoxic conditioning refers to the treatment of mice with intermittent hypoxia during long‐term hypoxia. First, the hypoxic chamber was set to a continuous low oxygen concentration of 13% O_2_ for 7 and 14 d. Second, intermittent hypoxia parameters were added in the hypoxia treatment process; four modes (Figure 3A) of intermittent hypoxic conditioning were used in this study. Mode①: 5 min 7% O_2_ hypoxic concentration and 5 min 13% O_2_ hypoxic concentration, a total of 10 cycles. Mode②: 5 min 10% O_2_ hypoxic concentration and 5 min 13% O_2_ hypoxic concentration, a total of 10 cycles. Mode③: 5 min 21% O_2_ concentration and 5 min 13% O_2_ hypoxic concentration, a total of 10 cycles. Mode④: 5 min 30% O_2_ concentration and 5 min 13% O_2_ hypoxic concentration, a total of 10 cycles. Each intermittent hypoxic mode lasted about 2 h per d. After treatment, the mice were removed and animal behavior experiments were conducted to observe whether the four modes showed antibrain injuries induced by long‐term continuous hypoxia.

### 2.3 Open field test (OFT)

The OFT was conducted in a quiet and poorly lit environment. Each animal was first placed in the right corner of the bottom of a box (the length, width, and height of the box were 40, 40, and 40 cm, respectively). It should be noted that to ensure that the test results were not affected by urine and feces left by previously housed animals, the inner wall and bottom of the box were thoroughly cleaned and sprayed with alcohol to remove any odors between each test. Animal activity was monitored for 5 min using the SMART Behavior Analysis System (Panlab, Barcelona, Spain). Each animal was acclimated in the open field for 5 min before being tested.

### 2.4 Morris water maze (MWM)

The water maze test used in this study was comprised of a circular tank that was 120 cm in diameter with a platform. The tank was filled with 23 ± 2°C tap water. Different shapes and colors were posted along the curtains of the tank, which served as spatial reference cues. A camera was mounted above the tank to record swimming tracks in the water maze. During the acquisition trials lasting 5 d, the platform was submerged 1–2 cm below the water surface, and the mice were placed into the maze at one of four quadrants facing the curtains of the tank. Mice were allowed 60 s to search for the platform. If mice failed to find the platform, they were guided to the platform and held on the platform for 5 s. Four trials per day were conducted with a minimum intermission of 1 h between trials. Escape latency indicative of spatial memory acquisition was recorded during each trial. On day 6, the platform was removed, a probe test was conducted, and the mice were placed in the water at one quadrant (usually the farthest quadrant from the hidden platform). Each mouse swam in the water for 60 s, during which the first attempt at finding the location, as well as the number of times crossing the platform, were recorded.

### 2.5 Novel object recognition (NOR)

The experimental device was 40 cm in length, 40 cm in width, and 40 cm in height. The experiment was divided into three stages, including conditioning, familiarity, and testing. The identifications of objects A and B were performed for two different materials and colors. In the adaptive phase, there was no object in the recognition apparatus, and the mice were given 5 min to adapt to the environment of the box. In the training phase, there were two identical instances of object A (two identical black square boxes) in the recognition apparatus, and the mice were placed in a familiar location in the environment for 5 min. In the testing phase, one instance of object A was replaced with object B (a white ball) in the recognition apparatus, and the video tracing system recorded the time required for mice to identify object B. The recognition index (RI) was calculated using the formula: RI = (N − F)/(N + F) to evaluate the ability of mice to recognize the novel object, where N (new) represented the total time required for the mouse's nose to touch object B, and F (former) represented the total time required for the mouse's nose to touch object A.

### 2.6 Rotarod test (RT)

The initial speed of the rotary rod fatigue meter was adjusted to 4 rpm, and was uniformly accelerated to the maximum speed of 40 rpm within 5 min. Five mice were then simultaneously placed on the rotating rod. The mice were trained 3 d before the formal test, three times a day for 5 min each time, with an interval of 30 min for each mouse. During the formal test, with 4–40 rpm uniform acceleration within 5 min, three tests were performed for 5 min each, with each mouse tested 30 min apart. The final results were averaged, and the time spent by the mouse on the rotating rod was recorded.

### 2.7 The 5′‐bromo‐2‐deoxyuridine (BrdU) injection

BrdU was obtained from Sigma‐Aldrich (St. Louis, MO, USA) and dissolved to 10 mg/ml in 0.9% NaCl. For proliferation assays, BrdU was injected intraperitoneally into mice five times at a dose of 50 mg/kg body weight at 1 d before collecting brain tissue.

### 2.8 TUNEL staining

For antigen repair, samples in citrate buffer (pH = 6.0) were boiled for 10 min and incubated with 1% PBST (phosphate buffered saline TritonX) at room temperature for 30 min. The ratio of enzyme to marker solution was 1:9. Samples were then incubated in a wet box at 37°C for 60 min in the dark. The slices were then washed three times and stained with diamidino‐2‐phenylindole (DAPI). At least three animals were stained in each group, and at least two brain slices were stained in each group; the thickness of each slice was 40 μm. TUNEL positive cells in the hippocampal region were counted after each brain slice was analyzed by confocal microcopy and photographed using a 630× magnification (Objective, 63× magnification and eyepiece with 10× magnification).

### 2.9 BrdU Staining

For antigen repair, samples in citric acid buffer (pH = 6.0) were boiled for 10 min, then washed with 0.01 M phosphate‐buffered saline (PBS), with shaking, three times, 10 min each time, followed by incubation with 1% PBST at room temperature for 30 min. Following this, samples were then washed three times with shaking in 0.01 M PBS for 10 min each time. For DNA degeneration, a mixture of 50% formamide and 2 × SSC was incubated at 65°C for 2 h, then with 2 × SSC, and incubated for 10 min.

After an incubation in 2 N HCl at 37°C for 30 min, borate buffer was used to wash four times, for 10 min each wash. Blocking used a 3% bovine serum albumin with a 1 h incubation at room temperature. The primary antibody incubation involved BrdU (Abcam, Ab220074) diluted 1:500, incubated at 4°C for 36 h, then three washes with 0.01 M PBS with shaking for 10 min each time. The secondary antibody incubation used Alexa Flour 488‐labeled (Invitrogen, Carlsbad, CA, USA) antibody with a 1:500 dilution, incubated for 2 h at room temperature in the dark. After staining with DAPI, a fluorescence microscope was used for observation, and representative photographs were captured, followed by statistical analysis using GraphPad software (GraphPad, San Diego, CA, USA). BrdU, positive cells were analyzed by confocal microcopy.

### 2.10 Immunofluorescence

Antigen repair, samples in citric acid buffer (pH = 6.0) were boiled for 10 min, then washed with 0.01 M phosphate‐buffered saline (PBS), with shaking, three times, 10 min each time, followed by incubation with 1% PBST at room temperature for 30 min. Following this, samples were then washed three times with shaking in 0.01 M PBS for 10 min each time. Blocking used a 3% bovine serum albumin with a 1 h incubation at room temperature. The primary antibody incubation involved PCNA (or DCX, Iba1, SOX2) diluted 1:500, incubated at 4°C for 36 h, then three washes with 0.01 M PBS with shaking for 10 min each time. The secondary antibody incubation used Alexa Flour 488‐labeled antibody with a 1:500 dilution, incubated for 2 h at room temperature in the dark. After staining with DAPI, a fluorescence microscope was used for observation, and representative photographs were captured. All positive cells were analyzed by confocal microcopy.

SOX2 (Abcam, Ab264112); DCX (Servicebio, GB113695); PCNA (Servicebio, GB11010); Iba1 (Servicebio, GB11105).

### 2.11 RT‐PCR

An RNeasy kit (Qiagen, Hilden, Germany) was used to extract total RNA from mice hippocampal tissue, and then the Transcriptor High Fidelity cDNA synthesis kit (Roche, Indianapolis, IN, USA) was used to reverse transcribe the RNA into cDNA. All methods were performed according to the manufacturer's instructions. The following primers were used: mouse β‐actin, forward: 5′‐GGCTGTATTCCCCTCCATCG‐3′, reverse: 5′‐CCAGTTGGTAACAATGCCATGT‐3′; mouse IL‐1β, forward: 5′‐ GCATCCAGCTTCAAATCTCGC‐3′, reverse 5′‐ TGTTCATCTCGGAGCCTGTAGTG‐3′; mouse IL‐6, forward: 5′‐ CCCCAATTTCCAATGCTCTCC‐3′, reverse 5′‐ CGCACTAGGTTTGCCGAGTA‐3′; and mouse TNF‐α, forward: 5′‐ CCCTCACACTCACAAACACC‐3′, reverse 5′‐ CTTTGAGATCCATGCCGTTG‐3′.

### 2.12 Statistics

The data are expressed as the mean ± SD. Statistical analyses were conducted using Prism 9.0 software (GraphPad). Statistical significance was assessed using Student's *t*‐tests or one‐way analysis of variance. A value of *p* < 0.05 was assumed to be statistically significant.

## 3 RESULTS

### 3.1 Long‐term hypoxia induced brain morphological and functional defects in mice

To better simulate environmental hypoxia, we chose 13% O_2_ (oxygen concentration at an altitude of approximately 3700 m) to continuously treat mice for 1, 3, 7, and 14 d (Figure 1A). The OFT and RT tests were used to characterize the motor behavior of mice, and the NOR and MWM tests were used to characterize the cognitive behavior of mice.

The OFT and RT results demonstrated that short‐term hypoxia did not cause abnormal motor behavior in mice, while long‐term hypoxia caused abnormal motor behavior in mice. Using the OFT, mice subjected to hypoxic treatment for 1 and 3 d (H1d and H3d) showed no significant behavioral differences when compared with the control, while mice subjected to hypoxic treatment for 7 and 14 d (H7d and H14d) showed lower times and distances in the center region (%), indicating that the mice had anxiety and abnormal spontaneous activities (Figure 1B–F). Using the RT, mice in the H7d and H14d groups showed impaired balance and movement compared with the control mice (Figure 1G). Regarding cognitive behavior, the NOR results suggested that mice in the H7d and H14d groups showed a lower discrimination index compared with the control mice, indicating that their memories were impaired (Figure 1H). The MWM results also showed that H7d and H14d mice had decreased spatial memories compared with the control group (Figure 1I–K).

Using TUNEL staining (Figure 1L,M), TUNEL‐positive cells were almost absent in hippocampal CA1 neurons of mice in the normal control group, and H1d and H3d groups, while TUNEL‐positive cells in the hippocampal CA1 neurons of mice in the H7d and H14d groups were significantly increased, suggesting that long‐term hypoxia induced increased hippocampal neuron apoptosis in mice. Taken together, these results indicated that short‐term hypoxia did not impair neurological function, while long‐term chronic hypoxia caused severe hypoxic responses (impaired neurological function and increased hippocampal neuron apoptosis) in mice.

### 3.2 Hypoxia promotes neurogenesis in a time‐dependent manner

**FIGURE 2:**
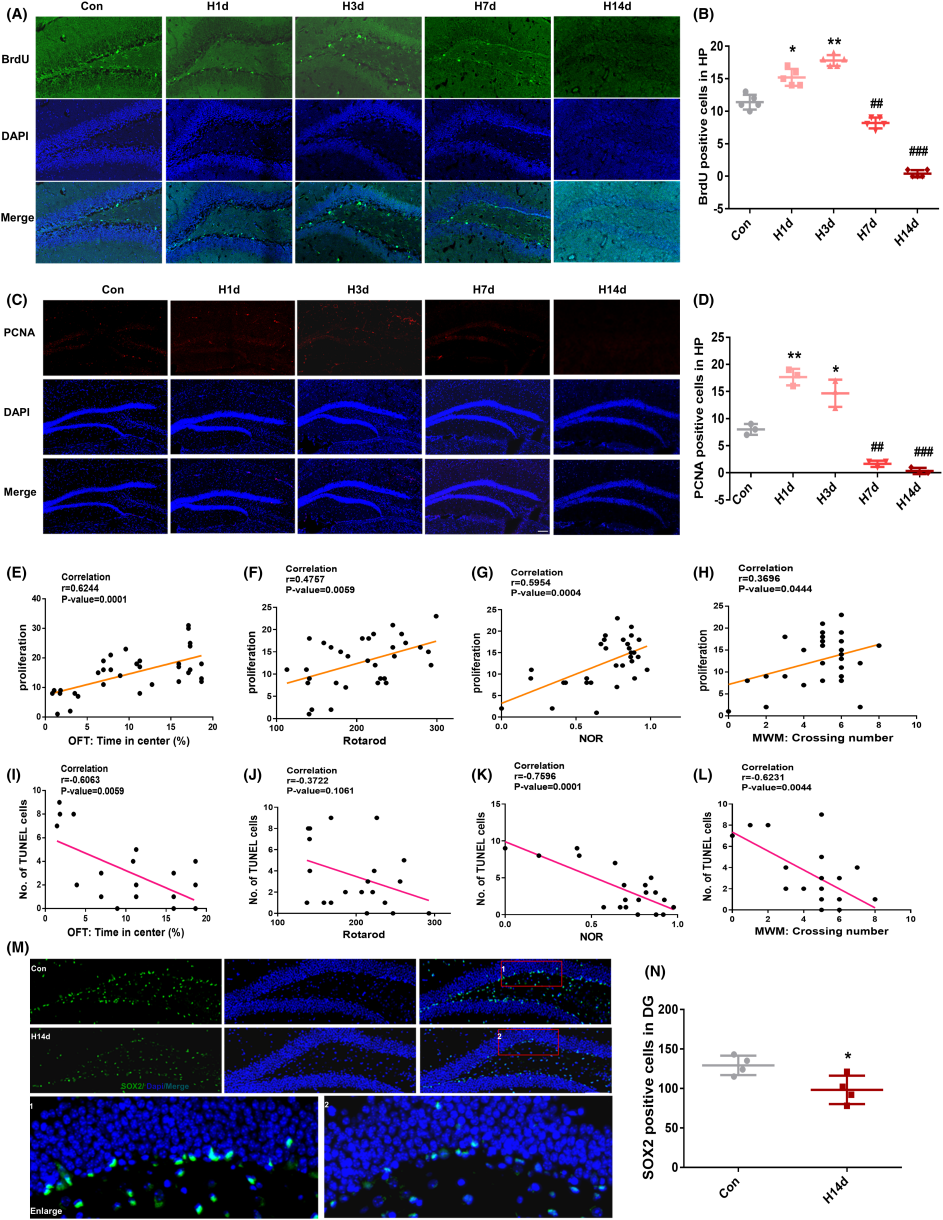
Short‐term hypoxia promotes neurogenesis and long‐term hypoxia inhibits neurogenesis by depleting neural stem cells. (A, B) BrdU staining and the count of BrdU‐positive cells in the dentate gyrus (DG) of hippocampus, Bar = 50 μm. (C, D) PCNA staining in the hippocampus of mice. And histograms showing the number of PCNA‐positive cells as shown in A, Bar = 50 μm. (E–H) Neurogenesis was positively correlated with animal behavior. (I–L) Apoptosis was negatively correlated with animal behavior. (M, N) SOX2 staining and the count of SOX2‐positive cells in DG of hippocampus. All data are expressed as the mean ± SD; *p < 0.05; **p < 0.01 versus the Con group. ^#^ *p* < 0.05 versus the H14d group.

To characterize the effects of hypoxia on neurogenesis, BrdU and proliferating cell nuclear antigen (PCNA) staining were performed. The BrdU staining showed that, compared with the mice in control group, the BrdU‐positive cells increased in the hippocampus H1d and H3d mice, but significantly decreased in the hippocampus H7d and H14d mice (Figure 2A,B). Similar results were also seen in the PCNA staining, compared with the control group, the PCNA‐positive cells increased in the hippocampus of the H1d and H3d groups, but decreased in the H7d and H14d groups (Figure 2C,D). Together, these results confirm that hypoxia regulates hippocampal neurogenesis bidirectionally in a time‐dependent manner.

Overall, these results suggested that hypoxia played a bidirectional role in the regulation of neurogenesis, which was mainly modulated by the duration of hypoxia.

### 3.3 Long‐term hypoxia induced depletion of neural stem cells in the hippocampus of mice and inhibited neurogenesis

To determine the relationship between neurogenesis and neurological functions in mice under hypoxia stress, we performed correlation analyses between neurogenesis, apoptosis, and neurological functions. The results of correlation analyses showed that motor and cognitive functions were associated with neurogenesis in mice, suggesting that neurogenesis was positively correlated with ethological performance when neurological function was impaired (Figure 2E–H). However, apoptosis was negatively correlated with neural function (Figure 2I–L).

To clarify what caused long‐term hypoxia to inhibit neurogenesis, we conducted SOX2 staining, which is a neural stem cell marker. In Figure 2M,N, when compared with the control group, the SOX2‐positive cells in the hippocampus of mice in the H14d group were significantly reduced, with a statistical difference, suggesting that long‐term hypoxia induced the depletion of neural stem cells in the hippocampus of mice. We, therefore, hypothesized that the inhibition of neurogenesis by long‐term hypoxia was due to the depletion of neural stem cells and the inability to generate new neurons in time to compensate for the apoptosis and degeneration, and necrosis induced by hypoxia, which ultimately led to neurological dysfunctions.

### 3.4 Intermittent hypoxic conditioning alleviates neurological dysfunction induced by long‐term hypoxia

**FIGURE 3:**
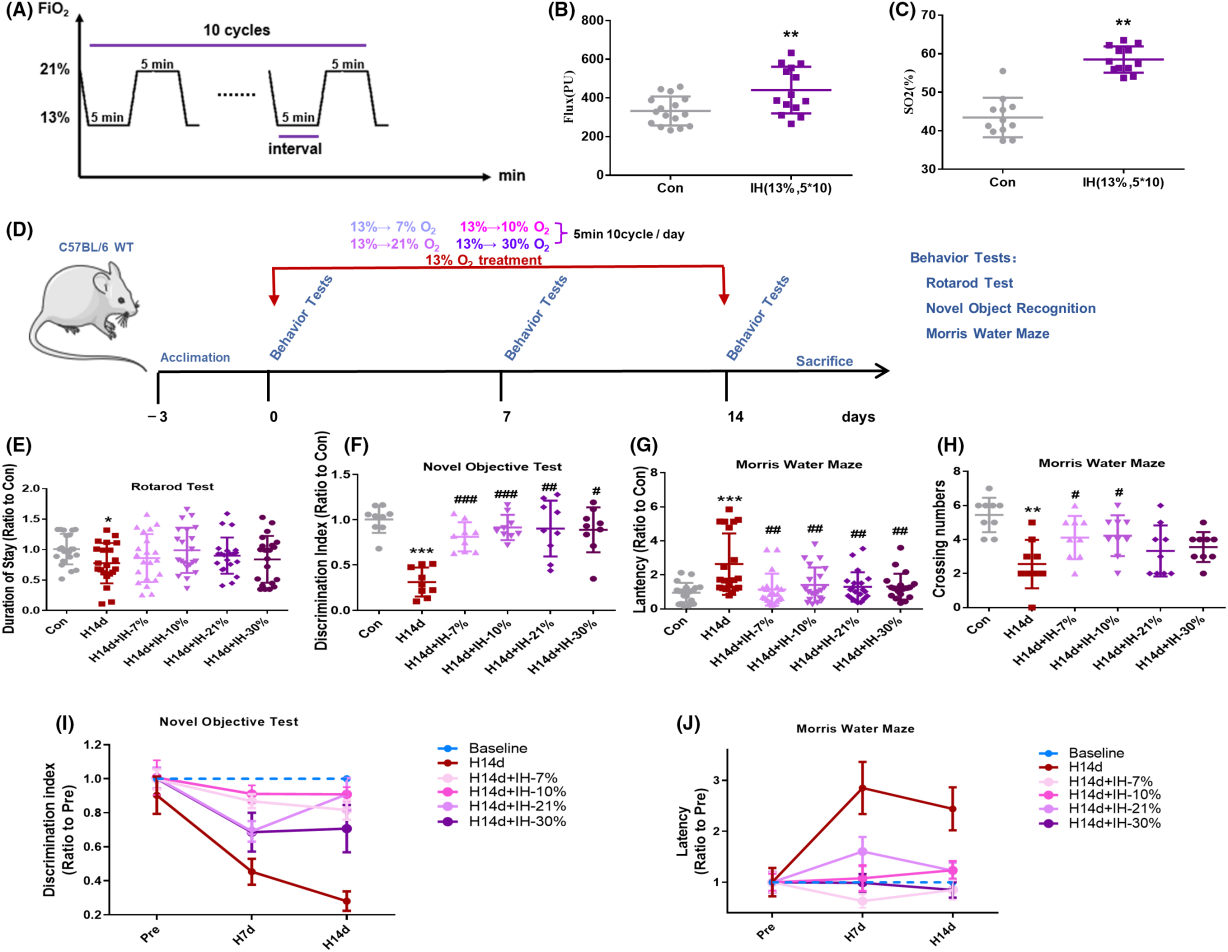
Experimental design and intermittent hypoxia conditioning restores behavioral deficits induced by long‐term hypoxia. (A) Pattern of intermittent hypoxia, 5 min 13% O_2_ and 5 min 21% O_2_ in total 10 cycles; (B) Intermittent hypoxia promoted increased flux in mice; (C) Intermittent hypoxia promoted the increase of SO_2_% in mice; (D) Schematic representation of the experimental design. The four intermittent hypoxic mode phase conditionings include Mode①: 5 min 7% O_2_ and 5 min 13% O_2_, a total of 10 cycles. Mode②: 5 min 10% O_2_ and 5 min 13% O_2_, a total of 10 cycles. Mode③: 5 min 21% O_2_ and 5 min 13% O_2_, a total of 10 cycles. Mode④: 5 min 30% O_2_ and 5 min 13% O_2_, a total of 10 cycles. Behavioral tests were performed at 0, 7 and 14 d of hypoxia treatment. (E) The recordings of mice duration on the rotarod test. (F) The discrimination index of mice in novel object recognition. (G, H) The latency and the crossing numbers of mice were recorded in the Morris water maze test. (I, J) Comparison of the effects of four modes of intermittent hypoxia conditioning to alleviate cognitive impairment induced by long‐term hypoxia. Data are expressed as the mean ± SD; *p < 0.05; **p < 0.01 versus the Con group. ^#^ *p* < 0.05 versus the H14d group.

Intermittent hypoxia has been reported to have a neuroprotective effect. In this study, we first showed a pattern of intermittent hypoxia based on previous studies, as shown in Figure 3A–C, and found that 5 min hypoxia (13% O_2_) and 5 min normoxia (21% O_2_), for a total of 10 cycles of intermittent hypoxia, increased the cerebral blood flow (flux) and blood oxygen saturation (SO_2_%) of mice, suggesting that this pattern of intermittent hypoxia had neuroprotective effects. The mode of intermittent hypoxia in this study, therefore, involved 5 min of hypoxia and 5 min of normoxia in a total of 10 cycles. Intermittent hypoxia included preconditioning and phase conditioning. To determine a better effect of hypoxic conditioning and oxygen inhalation, four modes of intermittent hypoxic conditioning were selected (Figure 3D). Several animal behavior results (Figure 3E–G) showed that all four modes of intermittent hypoxic conditioning had different effects on the antichronic hypoxia‐induced neurological dysfunctions. After comparing the four modes of conditioning (Figure 3H–I), it was found that the 10% mode of conditioning had the best antichronic hypoxia‐induced neurological dysfunctions effect.

### 3.5 Intermittent hypoxic conditioning provides an antichronic hypoxic‐induced neurological injury effect by inhibiting inflammation

**FIGURE 4:**
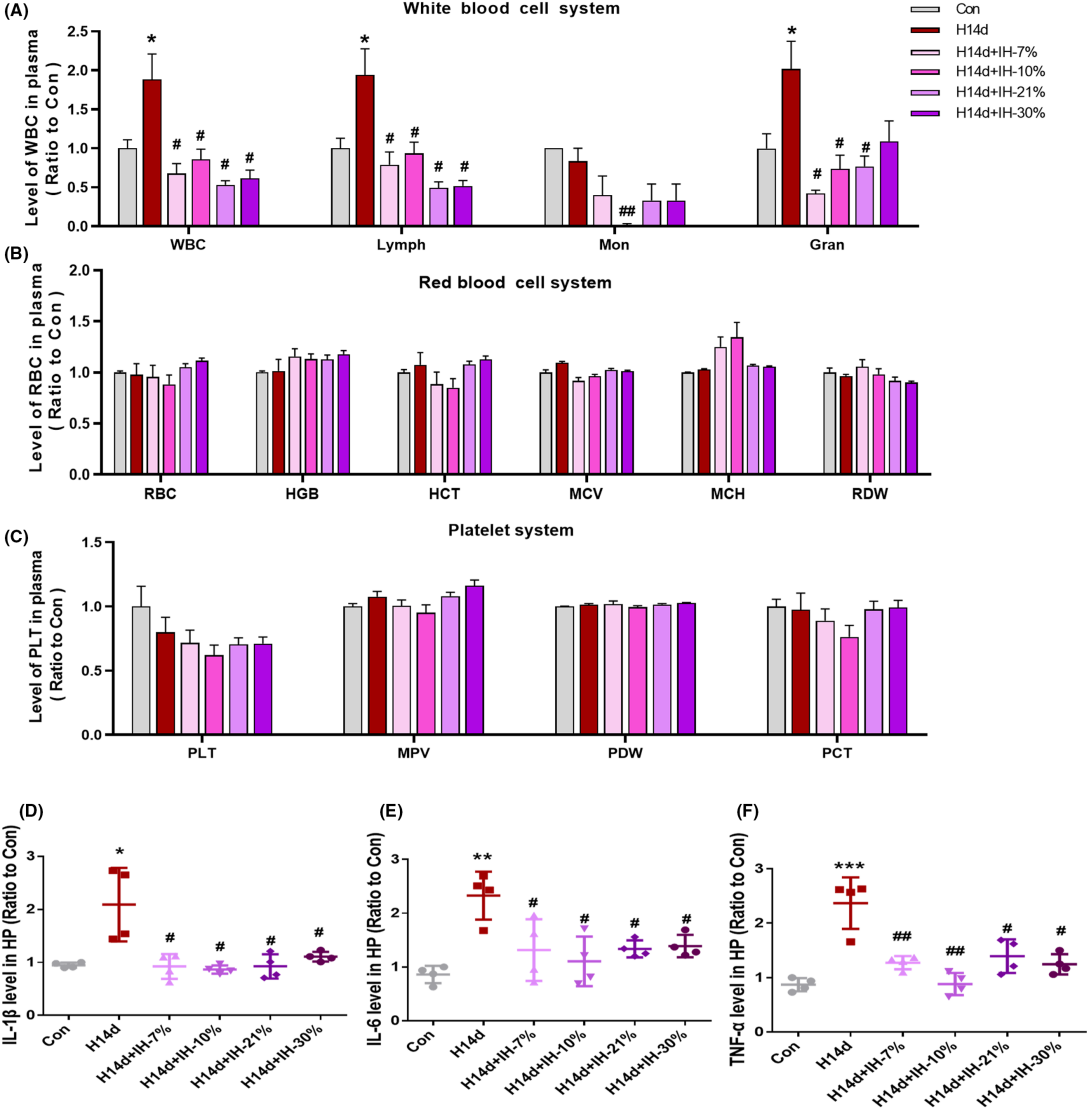
Intermittent hypoxia conditioning inhibits inflammatory stimulation in plasma and hippocampus of mice induced by long‐term hypoxia. (A) Intermittent hypoxic conditioning inhibited the elevation of leukocytes in plasma induced by long‐term hypoxia in mice. (B–D) Intermittent hypoxia conditioning had no effect on erythrocytes, platelet systems, and other biochemical indices in plasma of mice. (E, F) Intermittent hypoxia conditioning inhibits the release of inflammatory factors IL‐1β, IL‐6, and TNF‐ α in the hippocampus of mice. Data are expressed as the mean ± SD; *p < 0.05; **p < 0.01 versus the Con group. ^#^ *p* < 0.05 versus the H14d group.

To determine why intermittent hypoxia conditioning had an effect on neurologic dysfunctions induced by chronic hypoxia, we first examined changes in the blood cell components in the plasma of each group of mice. The results in Figure 4A–C showed that except for a white blood cell line, other biochemical tests involving a red blood cell line, platelet cell line, and triglyceride levels showed no significant changes, with no statistical differences. Figure 4A shows that the total number of white blood cells, lymphocytes, and neutrophils increased significantly in the H14d mice, when compared with the control group. However, the increases in the above cells were reversed in the four modes of intermittent hypoxia conditioning, showing statistical differences, suggesting that intermittent hypoxic conditioning played an important role in combating chronic hypoxic‐induced neurological dysfunctions, probably by inhibiting inflammation. To further confirm our hypothesis, we detected changes in inflammatory factors, such as IL‐1β, IL‐6, and TNF‐α, in the hippocampal tissues of each group of mice. The results are shown in Figure 4D–F. Compared with the control group, the expressions of IL‐1β, IL‐6, and TNF‐α in the hippocampus of mice in the H14d group were significantly increased, and the four modes of intermittent hypoxia conditioning reversed the increases of IL‐1β, IL‐6, and TNF‐α induced by long‐term hypoxia, showing statistical differences, suggesting that intermittent hypoxic conditioning played a role in antichronic hypoxic‐induced nerve injury, by inhibiting inflammation.

**FIGURE 5:**
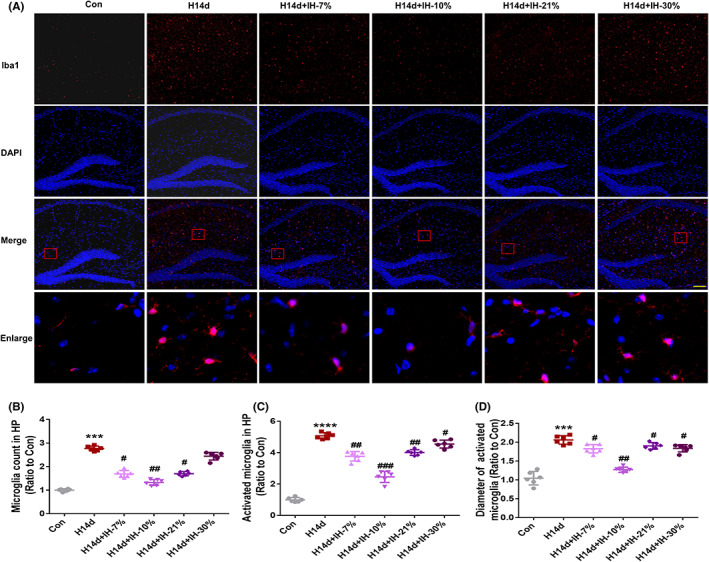
Intermittent hypoxia conditioning inhibited the proliferation and activation of microglia in mice hippocampus induced by long‐term hypoxia. (A) Intermittent hypoxia conditioning inhibited the proliferation and activation of microglia in mouse hippocampus induced by long‐term hypoxia. (B) Intermittent hypoxia conditioning inhibited the proliferation of microglia in mouse hippocampus induced by long‐term hypoxia. (C, D) Intermittent hypoxia conditioning inhibited the activation of microglia in mouse hippocampus induced by long‐term hypoxia. Data are expressed as the mean ± SD, bar = 50 μm; *p < 0.05; **p < 0.01 versus the Con group. ^#^ *p* < 0.05 versus the H14d group.

To further confirm this finding, we performed Iba1 staining to observe the number, morphology, and activation status of microglia in the hippocampal tissues of mice in each group. As shown in Figure 5A–D, compared with the control group, the total numbers of microglia and activated microglia in the hippocampus of mice in the H14d group were significantly increased, with a statistical difference. In addition, the morphology of activated microglia was amoeba‐like, and involved cell body hypertrophy, process atrophy. These results suggested that long‐term hypoxia induced inflammation in mice, while intermittent hypoxia conditioning, especially in the 10% group, had a significant anti‐inflammatory effect, which showed that the total number of microglia, the number of activated microglia, and the cell diameter of microglia were significantly reduced, when compared with H14d group, with a statistical difference, which is consistent with our previous ethological findings that the 10% model group had a better effect against long‐term hypoxia‐induced neurological dysfunctions. These observations provided further evidence that intermittent hypoxic conditioning played an important role in alleviating neuronal damage induced by long‐term hypoxia, by inhibiting inflammation.

### 3.6 Intermittent hypoxic conditioning alleviated long‐term hypoxia‐induced neurogenesis inhibition

**FIGURE 6:**
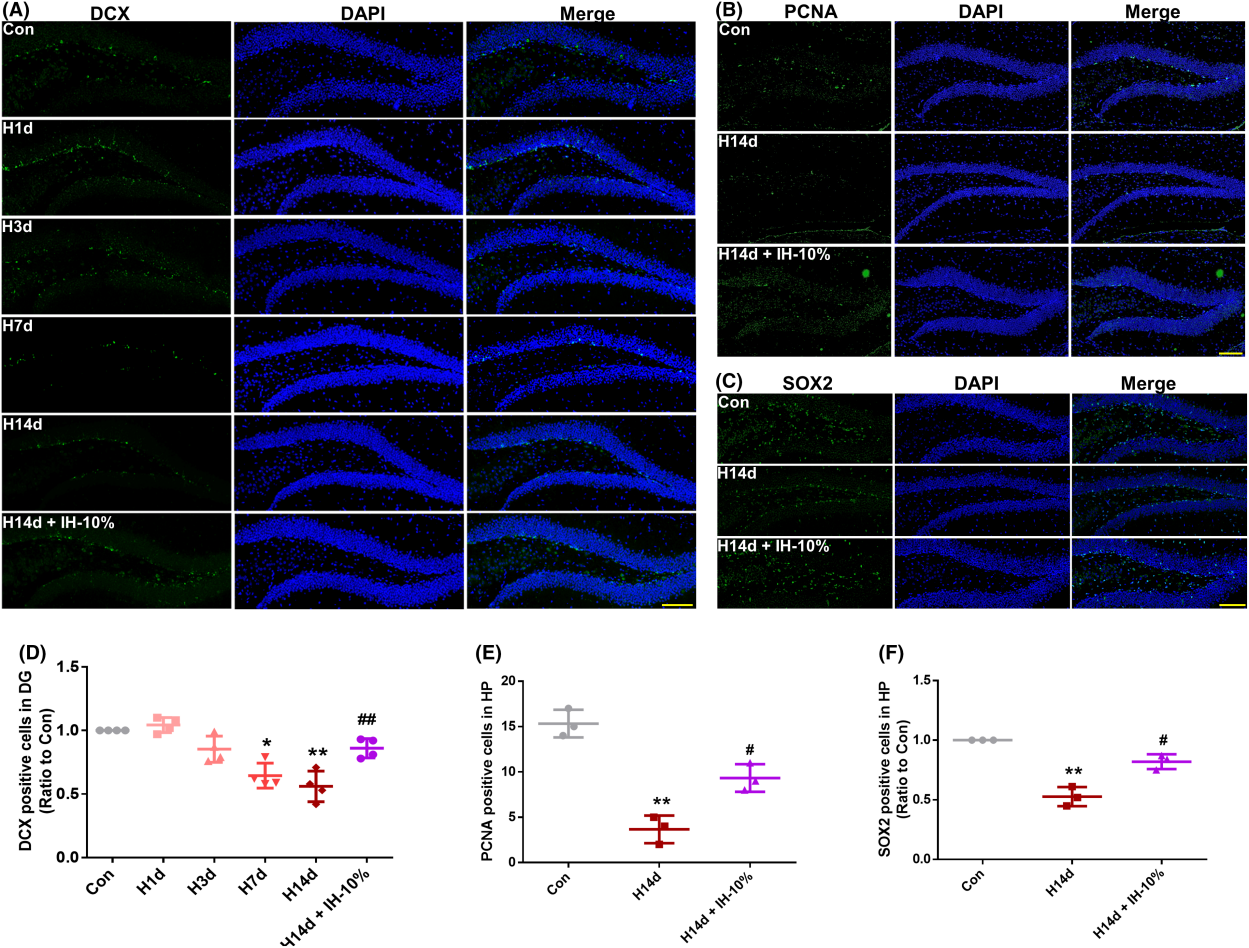
Intermittent hypoxia conditioning promoted the proliferation of neural stem cells which decreased induced by long‐term hypoxia. (A, D) DCX staining and the count of DCX‐positive cells in DG of hippocampus, Bar = 50 μm. (B, E) SOX2 staining and the count of SOX2‐positive cells in DG of hippocampus, Bar = 50 μm. (C, F) PCNA staining and the count of PCNA‐positive cells in DG of hippocampus, Bar = 50 μm.

In this study, we found that long‐term hypoxia inhibited neurogenesis and induced neurological dysfunction in mice. Intermittent hypoxic conditioning relieved long‐term hypoxia‐induced neurological dysfunction in mice by inhibiting inflammation, and the IH‐10% mode had the best effect. To explore the effects of intermittent hypoxic conditioning on neurogenesis, we performed PCNA, SOX2, and doublecortin (DCX) staining. The results showed that, compared with the control group, DCX‐positive cells significantly decreased in the hippocampus of H7d and H14d mice (Figure 6A,D), consistent with the previous BrdU and PCNA staining results, suggesting that long‐term hypoxia inhibits neurogenesis. However, compared with the H14d group, DCX‐positive cells in the hippocampus of H14d + IH‐10% mice increased significantly (Figure 6A,D). Meanwhile, in SOX2 and PCNA staining results also showed that, compared with the H14d group, SOX2‐ and PCNA‐positive cells in the hippocampus of H14d + IH‐10% mice increased significantly (Figure 6B,C,E,F). The above results suggested that intermittent hypoxic conditioning alleviated long‐term hypoxic‐induced neurogenesis inhibition by promoting proliferation of neural stem cells.

## 4 DISCUSSION

In this study, we found that mice tolerated or adapted to short‐term hypoxia stimulation with increased neurogenesis, but mice could not tolerate long‐term hypoxia stimulation with decreased neurogenesis. Several behavioral manifestations of mice were, therefore, correlated with neurogenesis, and it was found that there was a positive correlation between neurogenesis and the neurological functions of mice under certain conditions. However, this condition needs to be further explored, and we speculate that at a certain level of neurological impairment in mice, the less neurogenesis means the lower neurological functions of mice. To determine the reason about long‐term hypoxia in inhibiting neurogenesis, we further observed the changes in SOX2‐ and DCX‐positive cells (two neural stem cell marker) in the hippocampus of mice, and found that long‐term hypoxia led to the depletion of neural stem cells in mice. Therefore, we hypothesized that the failure of mice to tolerate long‐term hypoxia stimulation resulted from the obstruction of neurogenesis caused by the depletion of neural stem cells, and the inability to form new neurons to compensate for neuronal apoptosis and degeneration, and necrosis induced by hypoxic stimulation, resulting in severe neurological dysfunctions in mice.

In this study, the 13% O_2_ was used for low oxygen treatment, because the 13% O_2_ was close to the oxygen concentration at an altitude of about 3700 ms. Human activities at this altitude are not only common in China but also in the world.^28^, ^29^, ^30^ In addition, the hypoxic treatment times in this study were 0, 1, 3, 7, and 14 d, which was based on the conversion of human life expectancy from mice to human life expectancy, which is about 2 weeks for mice at the 14 d.^31^, ^32^ It has been reported in previous studies that people in the plain areas had cognitive dysfunctions after living on the plateau for 1–2 years.^33^, ^34^ In this study, neurological dysfunction was found in the H7d and H14d groups of mice, which is consistent with the results reported in previous studies. Therefore, the oxygen concentration (13% O_2_) and hypoxia treatment time (14 d) selected in this study were appropriate for the study of brain injuries induced by altitude hypoxia.

Previous studies found that intermittent hypoxia had a certain neuroprotective effect.^35^, ^36^ Even today, intermittent hypoxic training has been used extensively for the treatment of a variety of clinical disorders. In this study, we first characterized a pattern of intermittent hypoxia based on previous studies, using a mode of 5 min hypoxia and 5 min normoxia, in a total of 10 cycles. Intermittent hypoxic training includes preconditioning, phase conditioning, and postconditioning. Due to work requirements and time constraints, people traveling to high altitudes do not have time for intermittent hypoxic preconditioning, so this study mainly adopted the intermittent hypoxic conditioning model. Intermittent hypoxic conditioning involves treating people with lower levels of hypoxic stimulation or oxygen inhalation several cycles a day, while they are under hypoxia treatment. On the basis of previous studies, we selected four modes of intermittent hypoxic conditioning, Mode①: 5 min, 7% O_2_ and 5 min 13% O_2_, a total of 10 cycles. Mode②: 5 min 10% O_2_ and 5 min 13% O_2_, a total of 10 cycles. Mode③: 5 min 21% O_2_ and 5 min 13% O_2_, a total of 10 cycles. Mode④: 5 min 30% O_2_ and 5 min 13% O_2_, a total of 10 cycles.^37^ The results showed that all four models involved antihypoxic‐induced neurological dysfunctions, with the 10% mode working the best. However, the pathways through which these four modes of intermittent hypoxia conditioning play their role in antihypoxia‐induced neurological dysfunctions are not known. To further characterize this mechanism, we first performed a test on the plasma of each group of mice, and found that both hypoxia and intermittent hypoxia conditioning had no significant effect on the erythrocyte system, platelet system, and other biochemical parameters, but had the greatest effect on white cell parameters. We, therefore, hypothesized that intermittent hypoxia conditioning played an antihypoxia‐induced neural injury role by inhibiting inflammatory pathways. Therefore, in the later stage of this study, the changes in inflammatory factors and microglia in each group were observed. The results showed that intermittent hypoxia conditioning significantly inhibited long‐term hypoxia‐induced inflammatory factors and microglia proliferation. Microglia are equivalent to macrophages in the brain, and are the first and most important immune cells in the central nervous system.^38^ However, overactivation of microglia can induce an increase in inflammatory factors such as IL‐1β, IL‐6, and TNF‐α, which can cause nerve damage.^39^ Activated microglia appear to be amoeba‐like with hypertrophic cell bodies.^40^, ^41^ The results of this study showed that microglia in the hippocampus of mice in the H14d group were significantly activated, showing amoeba‐like hypertrophy. The four modes of intermittent hypoxia conditioning all had anti‐inflammatory effects, but the 10% mode had the best effect, which was consistent with the behavioral results.

Recent clinical and preclinical evidence suggests an interplay between neuroinflammation and neurogenesis.^42^ The presence of various inflammatory components, such as immune cells and cytokines, play a role in regulating the survival, proliferation, and maturation of neural stem cells.^43^ It is believed that appropriate inflammatory environment may provide suitable microenvironment for neurogenesis. However, the release of large amounts of proinflammatory cytokines can damage nerve cells, thereby inhibiting neurogenesis.^24^ In the present study, it was found that chronic hypoxia lead to reduced neurogenesis after the release of proinflammatory factors in the hippocampus of mice. This is consistent with the previous reported results. However, the detailed mechanisms underlying the inflammatory response that initiates or inhibits neurogenesis remain unclear and need to be further studied. In this study, we found that the amount of Iba1 in the hippocampal tissue of mice in the H14d group was significantly increased when cognitive function was impaired and neurogenesis was decreased, while the cognitive function of mice in the H14d group after intermittent hypoxia treatment was restored, neurogenesis was increased, and the amount of Iba1 was decreased.

Hypoxia has multiple regulatory effects on neurogenesis. Moderate hypoxia treatment has revealed a remarkable improvement in cognition by driving neurogenesis.^44^, ^45^ While severe hypoxia contributes to cognitive impairment by inhibiting neurogenesis.^46^ Moreover, repeated hypoxia induces brain inflammation and cognitive dysfunction in zebrafish,^47^ which contrasts with our results in rodents as reported here. Further elucidation of the mechanistic differences in the results between the above‐mentioned studies could lead to the discovery of novel targets for brain protection. Therefore, to make good use of hypoxia is to master the proper degree of hypoxia and the duration of hypoxia. What's more, one limittion of the current study is that only male mice were used in our study. However, sexual dimorphism has been reported for brain structures and metabolism,^48^, ^49^, ^50^ cerebral blood flow regulation under pathological conditions,^51^ and postischemia neurovascular remodeling.^52^ We will compare the effect of repeated hypoxia between female and male animals in future studies.

In summary, this study demonstrated that hypoxia had a dynamic regulatory effect on neurogenesis in mice. However, long‐term hypoxia resulted in the depletion of hippocampal neural stem cells in mice and the loss of their role in promoting neurogenesis, resulting in neurological dysfunction in mice. In addition, intermittent hypoxic conditioning was found to have an effect of antihypoxia‐induced neuronal dysfunction by inhibiting inflammation. In short, in the study we found that hypoxia at 13% O_2_ was found to dynamically regulate neurogenesis, and intermittent hypoxia conditioning exert neuroprotective effect by suppressing inflammation through promoting proliferation of neural stem cells.

## FUNDING INFORMATION

This research was supported by the National Natural Science Foundation of China (Grant numbers: 82027802 and 32100925), the Beijing Nova Program (Grant number: Z211100002121038), the Beijing Hundred Thousand and Ten Thousand Talents Project (Grant number: 2019A36), and the Beijing Municipal Health Commission (Grant number: 303‐01‐005‐0019).

## CONFLICT OF INTEREST

The authors declare that they have no competing interests. Dr. Jia Liu was excluded from all editorial decision‐making related to the acceptance of this article for publication.

## Data Availability

We ensure that all data are available.
